# Supplementary material for: Role and mechanism of NCAPD3 in promoting malignant behaviors in gastric cancer
Source: Front Pharmacol. 2024 Apr 22;15:1341039. doi: 10.3389/fphar.2024.1341039 (PMC11070777; doi:10.3389/fphar.2024.1341039)
Supplement: Supplementary file 11 [file DataSheet2.ZIP › GSEA/Transcipt factor/my_analysis.Gsea.1599461814734/SETD1A_TARGET_GENES.html]

Details for gene set SETD1A\_TARGET\_GENES[GSEA]

|  || Dataset | filtered\_dataset.sample\_info.cls#WT\_versus\_NCAPD3\_MUT |
| Phenotype | sample\_info.cls#WT\_versus\_NCAPD3\_MUT |
| Upregulated in class | WT |
| GeneSet | SETD1A\_TARGET\_GENES |
| Enrichment Score (ES) | 0.27839926 |
| Normalized Enrichment Score (NES) | 2.0224764 |
| Nominal p-value | 0.0 |
| FDR q-value | 0.3188376 |
| FWER p-Value | 0.545 |
Table: GSEA Results Summary

  

Fig 1: Enrichment plot: SETD1A\_TARGET\_GENES      
 Profile of the Running ES Score & Positions of GeneSet Members on the Rank Ordered List

  

| SYMBOL | TITLE | RANK IN GENE LIST | RANK METRIC SCORE | RUNNING ES | CORE ENRICHMENT || 1 | 51111 | KMT5B | 2 | 1.217 | 0.0155 | Yes |
| 2 | 51312 | SLC25A37 | 12 | 1.077 | 0.0236 | Yes |
| 3 | 388536 | ZNF790 | 52 | 0.913 | 0.0059 | Yes |
| 4 | 6428 | SRSF3 | 55 | 0.907 | 0.0171 | Yes |
| 5 | 157680 | VPS13B | 78 | 0.838 | 0.0116 | Yes |
| 6 | 112840 | WDR89 | 96 | 0.798 | 0.0095 | Yes |
| 7 | 55006 | TRMT61B | 101 | 0.782 | 0.0174 | Yes |
| 8 | 7769 | ZNF226 | 125 | 0.777 | 0.0103 | Yes |
| 9 | 51643 | TMBIM4 | 129 | 0.773 | 0.0188 | Yes |
| 10 | 55781 | RIOK2 | 135 | 0.763 | 0.0256 | Yes |
| 11 | 55900 | ZNF302 | 145 | 0.740 | 0.0289 | Yes |
| 12 | 9848 | MFAP3L | 155 | 0.726 | 0.0321 | Yes |
| 13 | 51434 | ANAPC7 | 166 | 0.710 | 0.0342 | Yes |
| 14 | 23450 | SF3B3 | 178 | 0.697 | 0.0354 | Yes |
| 15 | 84190 | METTL25 | 195 | 0.684 | 0.0325 | Yes |
| 16 | 55798 | METTL2B | 199 | 0.678 | 0.0397 | Yes |
| 17 | 145389 | SLC38A6 | 202 | 0.673 | 0.0476 | Yes |
| 18 | 4836 | NMT1 | 208 | 0.669 | 0.0530 | Yes |
| 19 | 22948 | CCT5 | 215 | 0.663 | 0.0577 | Yes |
| 20 | 127253 | TYW3 | 225 | 0.654 | 0.0598 | Yes |
| 21 | 9931 | HELZ | 229 | 0.651 | 0.0666 | Yes |
| 22 | 84816 | RTN4IP1 | 232 | 0.649 | 0.0742 | Yes |
| 23 | 57521 | RPTOR | 239 | 0.645 | 0.0785 | Yes |
| 24 | 161779 | PGBD4 | 242 | 0.637 | 0.0859 | Yes |
| 25 | 51135 | IRAK4 | 245 | 0.635 | 0.0933 | Yes |
| 26 | 55745 | AP5M1 | 246 | 0.634 | 0.1022 | Yes |
| 27 | 92806 | CENPBD1 | 249 | 0.631 | 0.1095 | Yes |
| 28 | 87178 | PNPT1 | 263 | 0.622 | 0.1080 | Yes |
| 29 | 25973 | PARS2 | 266 | 0.620 | 0.1152 | Yes |
| 30 | 116143 | WDR92 | 267 | 0.619 | 0.1239 | Yes |
| 31 | 10944 | C11orf58 | 272 | 0.617 | 0.1294 | Yes |
| 32 | 4238 | MFAP3 | 279 | 0.609 | 0.1333 | Yes |
| 33 | 80124 | VCPIP1 | 282 | 0.607 | 0.1402 | Yes |
| 34 | 84078 | KBTBD7 | 295 | 0.594 | 0.1392 | Yes |
| 35 | 25972 | UNC50 | 297 | 0.593 | 0.1467 | Yes |
| 36 | 25852 | ARMC8 | 307 | 0.588 | 0.1479 | Yes |
| 37 | 147929 | ZNF565 | 311 | 0.587 | 0.1538 | Yes |
| 38 | 7014 | TERF2 | 323 | 0.579 | 0.1533 | Yes |
| 39 | 84135 | UTP15 | 330 | 0.576 | 0.1567 | Yes |
| 40 | 51491 | NOP16 | 334 | 0.573 | 0.1624 | Yes |
| 41 | 56910 | STARD7 | 335 | 0.573 | 0.1705 | Yes |
| 42 | 4297 | KMT2A | 337 | 0.573 | 0.1778 | Yes |
| 43 | 5356 | PLRG1 | 338 | 0.573 | 0.1858 | Yes |
| 44 | 55676 | SLC30A6 | 353 | 0.565 | 0.1828 | Yes |
| 45 | 2965 | GTF2H1 | 356 | 0.563 | 0.1891 | Yes |
| 46 | 115509 | ZNF689 | 374 | 0.551 | 0.1836 | Yes |
| 47 | 64327 | LMBR1 | 378 | 0.547 | 0.1889 | Yes |
| 48 | 9786 | KIAA0586 | 379 | 0.547 | 0.1966 | Yes |
| 49 | 147007 | TMEM199 | 410 | 0.533 | 0.1806 | Yes |
| 50 | 54994 | GID8 | 415 | 0.526 | 0.1848 | Yes |
| 51 | 63929 | XPNPEP3 | 416 | 0.526 | 0.1922 | Yes |
| 52 | 57727 | NCOA5 | 425 | 0.517 | 0.1932 | Yes |
| 53 | 22796 | COG2 | 445 | 0.507 | 0.1855 | Yes |
| 54 | 146691 | TOM1L2 | 472 | 0.490 | 0.1720 | Yes |
| 55 | 5245 | PHB | 481 | 0.486 | 0.1726 | Yes |
| 56 | 29101 | SSU72 | 496 | 0.475 | 0.1683 | Yes |
| 57 | 1479 | CSTF3 | 500 | 0.474 | 0.1726 | Yes |
| 58 | 6742 | SSBP1 | 501 | 0.472 | 0.1792 | Yes |
| 59 | 54920 | DUS2 | 509 | 0.470 | 0.1803 | Yes |
| 60 | 130502 | TTC32 | 517 | 0.465 | 0.1814 | Yes |
| 61 | 4091 | SMAD6 | 519 | 0.462 | 0.1871 | Yes |
| 62 | 83448 | PUS7L | 520 | 0.462 | 0.1936 | Yes |
| 63 | 132001 | TAMM41 | 524 | 0.460 | 0.1977 | Yes |
| 64 | 10478 | SLC25A17 | 525 | 0.460 | 0.2041 | Yes |
| 65 | 204 | AK2 | 529 | 0.458 | 0.2082 | Yes |
| 66 | 29090 | TIMM21 | 537 | 0.453 | 0.2091 | Yes |
| 67 | 84081 | NSRP1 | 538 | 0.453 | 0.2155 | Yes |
| 68 | 8195 | MKKS | 542 | 0.449 | 0.2194 | Yes |
| 69 | 55527 | FEM1A | 543 | 0.449 | 0.2258 | Yes |
| 70 | 1819 | DRG2 | 550 | 0.447 | 0.2273 | Yes |
| 71 | 9416 | DDX23 | 553 | 0.445 | 0.2320 | Yes |
| 72 | 78988 | MRPL57 | 557 | 0.441 | 0.2359 | Yes |
| 73 | 57579 | FAM135A | 577 | 0.436 | 0.2271 | Yes |
| 74 | 23008 | KLHDC10 | 578 | 0.435 | 0.2332 | Yes |
| 75 | 6835 | SURF2 | 582 | 0.434 | 0.2370 | Yes |
| 76 | 90806 | ANGEL2 | 583 | 0.434 | 0.2431 | Yes |
| 77 | 26057 | ANKRD17 | 589 | 0.431 | 0.2452 | Yes |
| 78 | 9653 | HS2ST1 | 591 | 0.429 | 0.2505 | Yes |
| 79 | 23195 | MDN1 | 596 | 0.426 | 0.2533 | Yes |
| 80 | 56339 | METTL3 | 613 | 0.417 | 0.2466 | Yes |
| 81 | 55308 | DDX19A | 615 | 0.416 | 0.2517 | Yes |
| 82 | 10445 | MCRS1 | 617 | 0.414 | 0.2567 | Yes |
| 83 | 22847 | ZNF507 | 618 | 0.413 | 0.2625 | Yes |
| 84 | 5147 | PDE6D | 624 | 0.410 | 0.2644 | Yes |
| 85 | 55735 | DNAJC11 | 625 | 0.409 | 0.2701 | Yes |
| 86 | 26953 | RANBP6 | 630 | 0.406 | 0.2727 | Yes |
| 87 | 11083 | DIDO1 | 631 | 0.406 | 0.2784 | Yes |
| 88 | 79807 | GSTCD | 644 | 0.402 | 0.2747 | No |
| 89 | 3145 | HMBS | 682 | 0.386 | 0.2511 | No |
| 90 | 23093 | TTLL5 | 684 | 0.386 | 0.2558 | No |
| 91 | 132949 | AASDH | 689 | 0.384 | 0.2580 | No |
| 92 | 89910 | UBE3B | 693 | 0.381 | 0.2610 | No |
| 93 | 10607 | TBL3 | 698 | 0.380 | 0.2632 | No |
| 94 | 23076 | RRP1B | 709 | 0.370 | 0.2606 | No |
| 95 | 23350 | U2SURP | 719 | 0.364 | 0.2587 | No |
| 96 | 83743 | GRWD1 | 720 | 0.364 | 0.2638 | No |
| 97 | 57466 | SCAF4 | 724 | 0.361 | 0.2665 | No |
| 98 | 58509 | CACTIN | 748 | 0.351 | 0.2534 | No |
| 99 | 51602 | NOP58 | 750 | 0.348 | 0.2575 | No |
| 100 | 10726 | NUDC | 754 | 0.346 | 0.2601 | No |
| 101 | 84148 | KAT8 | 757 | 0.344 | 0.2633 | No |
| 102 | 51118 | UTP11L | 773 | 0.336 | 0.2563 | No |
| 103 | 79081 | LBHD1 | 784 | 0.330 | 0.2531 | No |
| 104 | 9238 | TBRG4 | 786 | 0.330 | 0.2570 | No |
| 105 | 90231 | KIAA2013 | 787 | 0.330 | 0.2616 | No |
| 106 | 1973 | EIF4A1 | 804 | 0.319 | 0.2536 | No |
| 107 | 51575 | ESF1 | 812 | 0.309 | 0.2524 | No |
| 108 | 9221 | NOLC1 | 816 | 0.307 | 0.2544 | No |
| 109 | 9343 | EFTUD2 | 818 | 0.305 | 0.2579 | No |
| 110 | 84823 | LMNB2 | 833 | 0.293 | 0.2510 | No |
| 111 | 2224 | FDPS | 841 | 0.273 | 0.2494 | No |
| 112 | 23435 | TARDBP | 842 | 0.272 | 0.2532 | No |
| 113 | 6520 | SLC3A2 | 878 | -0.290 | 0.2299 | No |
| 114 | 23731 | TMEM245 | 891 | -0.306 | 0.2248 | No |
| 115 | 23412 | COMMD3 | 894 | -0.312 | 0.2276 | No |
| 116 | 440026 | TMEM41B | 932 | -0.355 | 0.2036 | No |
| 117 | 11077 | HSF2BP | 1008 | -0.408 | 0.1506 | No |
| 118 | 53349 | ZFYVE1 | 1018 | -0.417 | 0.1494 | No |
| 119 | 7871 | SLMAP | 1038 | -0.435 | 0.1407 | No |
| 120 | 253959 | RALGAPA1 | 1066 | -0.456 | 0.1259 | No |
| 121 | 100534599 | ISY1-RAB43 | 1082 | -0.462 | 0.1207 | No |
| 122 | 9939 | RBM8A | 1093 | -0.474 | 0.1195 | No |
| 123 | 5565 | PRKAB2 | 1094 | -0.474 | 0.1261 | No |
| 124 | 604 | BCL6 | 1136 | -0.504 | 0.1011 | No |
| 125 | 831 | CAST | 1160 | -0.524 | 0.0905 | No |
| 126 | 7518 | XRCC4 | 1175 | -0.540 | 0.0871 | No |
| 127 | 10001 | MED6 | 1190 | -0.561 | 0.0840 | No |
| 128 | 7994 | KAT6A | 1279 | -0.666 | 0.0245 | No |
| 129 | 83931 | STK40 | 1281 | -0.669 | 0.0331 | No |
| 130 | 493861 | EID3 | 1315 | -0.729 | 0.0175 | No |
| 131 | 219539 | YPEL4 | 1354 | -0.803 | -0.0010 | No |
| 132 | 8867 | SYNJ1 | 1387 | -0.979 | -0.0123 | No |
| 133 | 6541 | SLC7A1 | 1388 | -0.981 | 0.0015 | No |
| 134 | 6782 | HSPA13 | 1394 | -1.064 | 0.0125 | No |
Table: GSEA details [plain text format]

  

Fig 2: SETD1A\_TARGET\_GENES      
 Blue-Pink O' Gram in the Space of the Analyzed GeneSet

  

Fig 3: SETD1A\_TARGET\_GENES: Random ES distribution      
 Gene set null distribution of ES for **SETD1A\_TARGET\_GENES**
